# Supplementary material for: Impacts of hydropower on the habitat of jaguars and tigers
Source: Commun Biol. 2021 Dec 9;4:1358. doi: 10.1038/s42003-021-02878-5 (PMC8660786; doi:10.1038/s42003-021-02878-5)
Supplement: Supplementary file 2 — Supplementary Information [file 42003_2021_2878_MOESM2_ESM.pdf]

Supplementary Information

Impacts of hydropower on the habitat of jaguars and tigers

Ana Filipa Palmeirim, Luke Gibson

This Supplementary Information includes: Supplementary Figures 1 to 3, and Supplementary Table 1. Supplementary Data 1 to 3 can be found in the corresponding excel files.

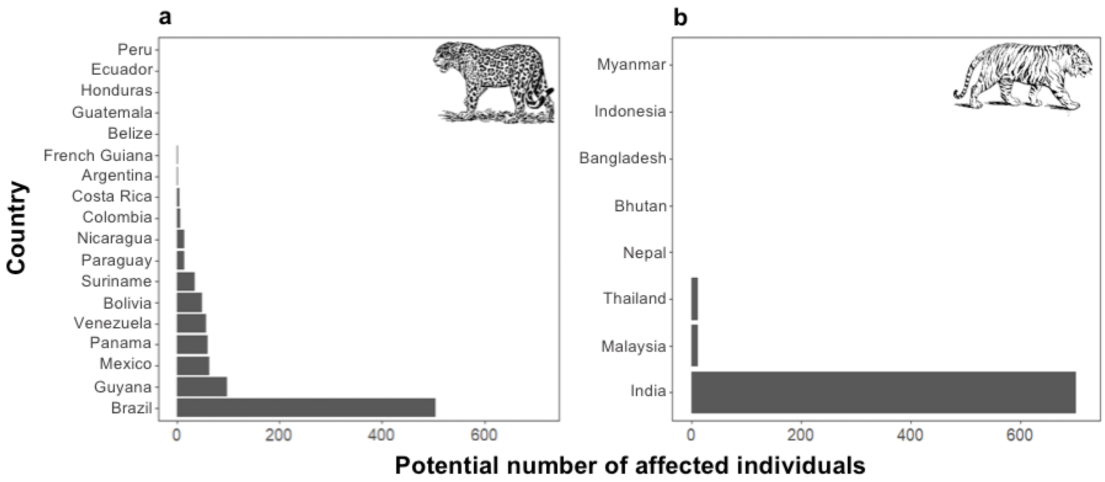

Supplementary Figure 1. Number of **a** jaguars and **b** tigers potentially affected due to habitat flooding following hydropower, by country. These figures were obtained by associating available species density values with reservoir area. Information on tiger and jaguar density values used for each reservoir can be found in Supplementary Data 1 and 2, respectively.

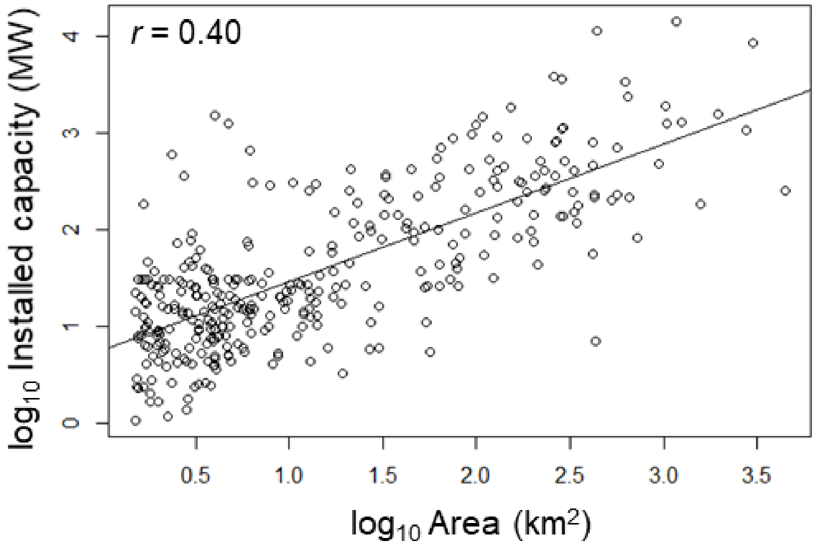

Supplementary Figure 2. Correlation between reservoir area ($\log_{10}$ x + 1; km^2) and installed capacity (MW; $\log_{10}$ x + 1) for existing and planned reservoirs intersecting jaguar distribution in Brazil.

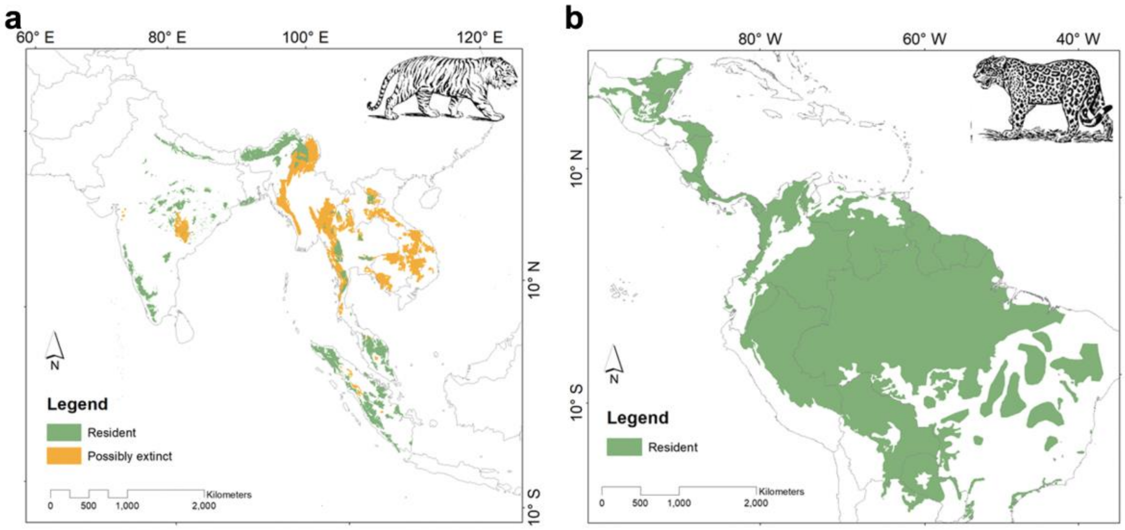

Supplementary Figure 3. Contemporary geographic distribution of **a** tigers and **b** jaguars (provided by IUCN spatial data). Tiger distribution includes both areas where tigers are resident and possibly extinct, but not an additional remaining population in the Russia-China border that was not considered in this study. Both maps are presented at the same scale for comparative purposes.

Supplementary Table 1. Sources used to extract information on dams for each country intersecting tiger and jaguar ranges.

Country	Source of information
Jaguar	
Argentina	FAO[1], Major dams[2]
Belize	FAO
Bolivia	FAO, Anderson et al. 2018[3], Latrubesse et al. 2017[4], Major dams
Brazil	ANEEL[5], Major dams, Fearnside (2015)[6]
Colombia	FAO, Anderson et al. 2018, Latrubesse et al. 2017
Costa Rica	FAO
Ecuador	FAO, Anderson et al. 2018, Latrubesse et al. 2017
French Guiana	FAO
Guatemala	CNEE[7], Lehner et al. 2011[8]
Guyana	FAO
Honduras	FAO
Mexico	FAO
Nicaragua	ENEL[9], FAO
Panama	FAO
Paraguay	FAO
Peru	Anderson et al. 2018, Latrubesse et al. 2017, Major dams
Suriname	FAO
Venezuela	FAO, Major dams
Tiger	
Bhutan	FAO, International Rivers (Bhutan)[10]
India	FAO, Lehner et al. 2011, International Rivers (India)[11], Major dams
Indonesia	FAO
Malaysia	FAO
Myanmar	Open Development Mekong[12]
Nepal	FAO, The 3rd pole[13]
Thailand	FAO, Open Development Mekong

Supplementary References

1. FAO. AQUASTAT website. Food and Agriculture Organization of the United Nations (FAO). Website accessed on [2018/06/01].
2. Major dams. State of the World's Rivers shared by Global Forest Watch. http://data.globalforestwatch.org/datasets/537361e2df59486e898cd4e024af57ea_0. Website accessed on [2018/06/01].
3. Anderson, E. P. *et al.* Fragmentation of Andes-to-Amazon connectivity by hydropower dams. *Sci. Adv.* 4, eaao1642 (2018).
4. Latrubesse, E. M. *et al*. Damming the rivers of the Amazon basin. *Nature* 546, 363 (2017).
5. ANEEL. Agência Nacional de Energia Elétrica (Brazil). http://www.aneel.gov.br. Website accessed on [2018/06/01].
6. Fearnside, P. M. Hidrelétricas na Amazônia: impactos ambientais e sociais na tomada de decisões sobre grandes obras-Volume 2 (2015).
7. CNEE. Comisión Nacional de Energía Eléctrica, República de Guatemala. http://www.cnee.gob.gt/. Website accessed on [2018/06/01].
8. Lehner, B., C. *et al.* Global Reservoir and Dam Database, Version 1 (GRanDv1): Reservoirs, Revision 01. Palisades, NY: NASA Socioeconomic Data and Applications Center (SEDAC). https://doi.org/10.7927/H4HH6H08 (2011).
9. ENEL. Empresa Nicaguarence de Electricidad: http://www.enel.gob.ni/. Website accessed on [2018/06/01].
10. International Rivers (Bhutan). Status of hydropower dams in Bhutan. https://www.internationalrivers.org/resources/8703. Website accessed on [2018/06/01].
11. International Rivers (India). Campaign for India. https://www.internationalrivers.org/campaigns/india. Website accessed on [2018/06/01].
12. Open Development Mekong Database. https://opendevelopmentmekong.net/search/data/. Website accessed on [2018/06/01].
13. The 3rd pole – Understanting Asia's water crisis (Ganges and Koshi Basins databases). https://www.thethirdpole.net. Website accessed on [2018/06/01].
14. Jędrzejewski, W. et al. Estimating large carnivore populations at global scale based on spatial predictions of density and distribution–Application to the jaguar (*Panthera onca*). *PloS One* 13, e0194719 (2018).
