# Supplementary material for: Impacts of hydropower on the habitat of jaguars and tigers
Source: Commun Biol. 2021 Dec 9;4:1358. doi: 10.1038/s42003-021-02878-5 (PMC8660786; doi:10.1038/s42003-021-02878-5)
Supplement: Supplementary file 3 — Description of Additional Supplementary Files [file 42003_2021_2878_MOESM3_ESM.pdf]

Description of Additional Supplementary Files

File name: Supplementary Data 1
Description: List of all dams intersecting the range of jaguars. For each dam, we included information on country, dam name, status (existing = E or planned = P), UTM geographic coordinates (North, East and Zone), reservoir area (km^2) and associated jaguar density and location. Jaguar density values were extracted from Jędrzejewski et al. 2018[14]. Locations from where density data were extracted are named as in Jędrzejewski et al. 2018[14]. The source of each dam compiled can be found in Supplementary Table 1.

File name: Supplementary Data 2
Description: List of all dams intersecting the range of tigers, including areas where tigers are resident (R) and other area where tigers are considered possibly extinct (PE). For each dam, we included information on country, dam name, status (existing = E or planned = P), UTM geographic coordinates (North, East and Zone), reservoir area (km^2) and associated tiger density (number of individuals per km^2), location and source from where tiger density values were extracted. The source of each dam compiled can be found in Supplementary Table 1.

File name: Supplementary Data 3
Description: Data used in the trade-off analysis between hydroelectricity generation and potential number of jaguars affected by existing (E) and planned (P) hydropower reservoirs. Jaguar density values were extracted from Jędrzejewski et al. 2018[14]. Regions from where density data were extracted are named as in Jędrzejewski et al. 2018[14]. The source of each dam compiled can be found in Supplementary Table 1. Although listed in this table, dams potentially affecting less than 0.0001 jaguars were excluded from the analysis.
