# Supplementary material for: Impacts of hydropower on the habitat of jaguars and tigers
Source: Commun Biol. 2021 Dec 9;4:1358. doi: 10.1038/s42003-021-02878-5 (PMC8660786; doi:10.1038/s42003-021-02878-5)
Supplement: Supplementary file 7 — Reporting Summary [file 42003_2021_2878_MOESM7_ESM.pdf]

nature research

Corresponding author(s): DBPR: COMMSBIO-21-1142-T

Last updated by author(s): Sep 1, 2021

Reporting Summary

Nature Research wishes to improve the reproducibility of the work that we publish. This form provides structure for consistency and transparency in reporting. For further information on Nature Research policies, see our Editorial Policies and the Editorial Policy Checklist.

Statistics

For all statistical analyses, confirm that the following items are present in the figure legend, table legend, main text, or Methods section.

n/a	Confirmed	
☐	☒	The exact sample size (*n*) for each experimental group/condition, given as a discrete number and unit of measurement
☐	☒	A statement on whether measurements were taken from distinct samples or whether the same sample was measured repeatedly
☐	☒	The statistical test(s) used AND whether they are one- or two-sided<br>*Only common tests should be described solely by name; describe more complex techniques in the Methods section.*
☐	☒	A description of all covariates tested
☐	☒	A description of any assumptions or corrections, such as tests of normality and adjustment for multiple comparisons
☐	☒	A full description of the statistical parameters including central tendency (e.g. means) or other basic estimates (e.g. regression coefficient) AND variation (e.g. standard deviation) or associated estimates of uncertainty (e.g. confidence intervals)
☐	☒	For null hypothesis testing, the test statistic (e.g. *F*, *t*, *r*) with confidence intervals, effect sizes, degrees of freedom and *P* value noted<br>*Give P values as exact values whenever suitable.*
☒	☐	For Bayesian analysis, information on the choice of priors and Markov chain Monte Carlo settings
☒	☐	For hierarchical and complex designs, identification of the appropriate level for tests and full reporting of outcomes
☐	☒	Estimates of effect sizes (e.g. Cohen's *d*, Pearson's *r*), indicating how they were calculated

Our web collection on statistics for biologists contains articles on many of the points above.

Software and code

Policy information about availability of computer code

Data collection	Google Earth Pro 5.2. DigitalGlobe 2019. http://www.earth.google.com.
Data analysis	R v3.6.3. R Development Core Team (2015) R: A language and environment for statistical computing. R Foundation for Statistical Computing, Vienna, Austria. http://www.R-project.org/.<br><br>ArcMap 10.1. ESRI, 2012. ArcMap 10.1. Environmental Systems Research Institute Inc., Redlands, CA, USA.

For manuscripts utilizing custom algorithms or software that are central to the research but not yet described in published literature, software must be made available to editors and reviewers. We strongly encourage code deposition in a community repository (e.g. GitHub). See the Nature Research guidelines for submitting code & software for further information.

Data

Policy information about availability of data

All manuscripts must include a data availability statement. This statement should provide the following information, where applicable:

- Accession codes, unique identifiers, or web links for publicly available datasets
- A list of figures that have associated raw data
- A description of any restrictions on data availability

All raw data used in this study are available in the supplementary material.

Field-specific reporting

Please select the one below that is the best fit for your research. If you are not sure, read the appropriate sections before making your selection.

☐ Life sciences ☐ Behavioural & social sciences ☒ Ecological, evolutionary & environmental sciences

For a reference copy of the document with all sections, see nature.com/documents/nr-reporting-summary-flat.pdf

Ecological, evolutionary & environmental sciences study design

All studies must disclose on these points even when the disclosure is negative.

Study description	We quantified the habitat loss following inundation of hydropower reservoirs across the distribution of jaguars and tigers. To do so, we compiled all existing and planned dams intersecting the distribution of these species. We also quantified the total habitat area flooded by reservoirs. In addition, we determined the ratio of the number of individuals lost per unit of energy produced (i.e., 100 MW) by existing and planned dams, we used data on the installed capacity and reservoir area of both existing and planned dams. As these data were only available for Brazil, where more than half of the total jaguar population remains, this last part of the analyses was carried out only for this species therein.
Research sample	Tiger and jaguar across their geographic ranges and all hydroelectric dams that intersect these species ranges.
Sampling strategy	We considered all existing and planned dams across tiger and jaguar ranges.
Data collection	We exhaustively searched for databases, published studies and reports including information on either existing or planned dams located in the current range states hosting tiger and jaguar populations. After catalogueing all dam information, we used the geographic coordinates provided by the source to overlap with the IUCN spatial data to identify those dams intersecting the current distribution of tigers and jaguars. For each dam, we collected information on location (geographic coordinates), status (existing or planned), reservoir area (km2) and installed capacity (MW). Whenever reservoir area was not available for existing dams, we manually measured it using Google Earth Pro. Dams were classified as (1) existing, if already in operation or under construction with known area; and (2) planned, if its construction has yet to begin (including both dams with and without studies/licensing completed), if its construction has begun but information on area is not available (suggesting its preliminary state of construction), or if its construction has been temporarily or permanently suspended, canceled, or revoked but that have the chance of being re-considered in future government.
Timing and spatial scale	Our data was compiled in 2019 and the spatial scale spans across the geographic range of tigers (i.e., India, Nepal, Myanmar, Malaysia, Indonesia, Bhutan, Bangladesh and Thailand) and jaguars (i.e., Peru, Ecuador, Honduras, Guatemala, Belize, French Guyana, Argentina, Costa Rica, Colombia, Nicaragua, Paraguay, Suriname, Bolivia, Venezuela, Panama, Mexico, Guyana and Brazil).
Data exclusions	Dams flooding less than 0.01 km2 were not included in further analyses. In addition, we did not include remnant tiger populations occurring in countries where this species is considered possibly extinct (i.e., Laos, Vietnam and Myanmar), as well as Russia and China due to the smaller number of remaining individuals therein (~400 ind.; Goodrich et al., 2015).
Reproducibility	All attempts to repeat the experiement were successful.
Randomization	We have compiled data on dams location, reservoir area and energy produced. We then crossed this information with the geographic range of tigers and jaguars. We then used density estimates for these species occurring nearby each dam to have an idea of how many individuals in total have hypothetically been affected by hydropower development. Thus, we study did not required randomization.
Blinding	Given all information provided above, blinding was not relevant for this study.

Did the study involve field work? ☐ Yes ☒ No

Reporting for specific materials, systems and methods

We require information from authors about some types of materials, experimental systems and methods used in many studies. Here, indicate whether each material, system or method listed is relevant to your study. If you are not sure if a list item applies to your research, read the appropriate section before selecting a response.

Materials & experimental systems

n/a	Involved in the study
☒	☐ Antibodies
☒	☐ Eukaryotic cell lines
☒	☐ Palaeontology and archaeology
☒	☐ Animals and other organisms
☒	☐ Human research participants
☒	☐ Clinical data
☒	☐ Dual use research of concern

Methods

n/a	Involved in the study
☒	☐ ChIP-seq
☒	☐ Flow cytometry
☒	☐ MRI-based neuroimaging
